# An Improved Measurement Method for the Strength of Radiation of Reflective Beam in an Industrial Optical Sensor Based on Laser Displacement Meter

**DOI:** 10.3390/s16050752

**Published:** 2016-05-23

**Authors:** Youngchul Bae

**Affiliations:** Department of Electrical and Semiconductor Engineering, Chonnam National University, Daehak-ro 50 Yeosu, Jeonnam 59626, Korea; ycbae@chonnam.ac.kr; Tel.: +82-61-659-7315

**Keywords:** laser ranger finder, optical sensor, reflect signal, return signal, radiation intensity, phase delay, strength of radiation

## Abstract

An optical sensor such as a laser range finder (LRF) or laser displacement meter (LDM) uses reflected and returned laser beam from a target. The optical sensor has been mainly used to measure the distance between a launch position and the target. However, optical sensor based LRF and LDM have numerous and various errors such as statistical errors, drift errors, cyclic errors, alignment errors and slope errors. Among these errors, an alignment error that contains measurement error for the strength of radiation of returned laser beam from the target is the most serious error in industrial optical sensors. It is caused by the dependence of the measurement offset upon the strength of radiation of returned beam incident upon the focusing lens from the target. In this paper, in order to solve these problems, we propose a novel method for the measurement of the output of direct current (DC) voltage that is proportional to the strength of radiation of returned laser beam in the received avalanche photo diode (APD) circuit. We implemented a measuring circuit that is able to provide an exact measurement of reflected laser beam. By using the proposed method, we can measure the intensity or strength of radiation of laser beam in real time and with a high degree of precision.

## 1. Introduction

An optical sensor such as a laser range finder (LRF) remotely measures distances by using the laser wavelength that is reflected and returned from a target. The optical sensor has been mainly used to measure the distance between a launch position and the target. Helicopters, tanks, and armored vehicles of military and defense forces have been equipped with them because the optical sensor comparatively provides exact measured distance to the target.

Typically, an optical sensor techniques based on laser displacement meter (LDM) can be divided into the following types: time-of-flight [1,2,3,4], triangulation [5,6], frequency modulation continuous-wave (FMCW) phase measurement [7,8], phase shift [9,10,11,12,13,14], interferometry method [15,16,17,18,19] and others [20,21,22]. Among these types, interferometry method is well-known for providing the highest resolution [15,16,17,18,19].

However, the optical sensor has a technical difficulty for making it into an industrial product because industrial products require more precise technique compared to products used in military. The measuring-distance range for the military is more than several km, and, in accordance with the precise military standard, the measuring-error parameters of the optical sensors are within 5 m to 10 m.

Although price unit of the optical sensor for military rather than for industry is much more expensive, there is no problem for the prices of laser and parts for the optical sensors in the military. However, the prices of the laser and its parts to manufacture the optical sensors are relatively expensive in industrial products compared to military products.

The efforts to transit the optical sensor into a commercial product, from the military to industry, has continued. To commercialize a product in the optical-sensor industry, it is necessary to manufacture a product with an increased precision, a high reliability, a low price, and a small size that is lightweight. The optical sensor can be applied in automated systems in the field including those commercial product that provide the distance measurements that protects a ship [23] from the damage as it is positioned alongside a pier, unmanned over-speed sensors and the collision-avoidance systems of vehicles [24]. Typically, an industrial optical sensor requires a measuring distance within 1 km and a measuring error within 1 mm to 10 mm to be compatible with industry, due to the characteristics that are required for unmanned industrial systems.

An optical sensor can measure precise distances using high re-flexibility and straight features, both of which are laser-beam characteristics. However, the strength of radiation of reflected laser beam at the target rapidly changes according to the distance and the surface status of the reflective object. As a consequence, the variation range of the measurement errors is largely influenced by the intensity (strong and weakness) of this reflected laser beam. In this case, to reduce measurement error, we have to greatly increase the number of measurements and then calculate the average value during a short time, within microseconds; as a result, the measurement-error variation range can be reduced. However, this is not reasonable because the measurement time is proportionally longer than the number of repeated measurements. Therefore, in a high-speed operation, a different method to reduce error is needed.

The errors in the industrial optical sensor are numerous and various. Many researchers focus on reduction of errors in the industrial optical sensor based on laser displacement meter. There are many sources of errors in the optical sensor such as statistical errors, drift errors, cyclic errors, alignment errors and slope errors in LRF or LDM. Among these errors, an alignment error is the most serious error in industrial optical sensors, and is caused by the dependence of the measurement offset upon the strength of radiation of returned beam incident upon the focusing lens from the target. The alignment error contains measurement error for the strength of radiation of laser beam.

Perchet *et al.* [14] proposed magnification of the phase-shift for LDM. This system uses two coherent indirect frequency synthesizers, with the reference signal and photoelectrical signal; however, this method also does not provide the circuit for error measurement for returned laser beam signal from target.

Hashemi *et al.* [20] studied the source of error in LRF, and they demonstrate several errors such as statistical error, drift error, cyclic error, alignment error and slope error. However, they did not propose the circuit for error measurement.

In order to measure the error for the strength of radiation of laser beam, we have to compensate the difference of phase delay in the APD. However, the optical demodulated signal for measuring are different from each other at the focusing lens, so it is hard to measure the strength of radiation of reflective laser beam. In this case, because the reflected signal level and phase difference in the target is not constantly reflected according to the strength of radiation of laser beam, variation factor of reflected signal level of laser beam take place according to the strength and amount of radiation of laser beam. In accordance with this variation factor, the error for result of measurement in LDM occurs. The variation factor of reflected signal level can largely be divided into two types. One is variation by surface re-flexibility in the target with identical distance. The other is that variation for distance differences exists in the target, even though they have the same surface re-flexibility. Therefore, we require a compensating technique for error by the variation of reflective signal level of laser beam in the industrial optical sensor.

In this paper, our sole focus is the reduction of the measurement error for the strength of radiation of returned laser beam from the target among alignment errors in the industrial optical sensor based on LDM. The reduction of measurement error in the industrial optical sensor is important to make a precise industrial optical sensor. This measurement error causes the phase delay that occurs in the APD output signal and its extent depends on the strength of the reflective laser beam that is returned from the target of the object. Even though this measurement error is very significant, an exact measuring-circuit technique to measure the error has not yet been developed. The difficulty of this situation is compounded because we are not only unable to measure an error, but also we cannot try to reduce the incidence of errors. To compensate for an error is difficult, because the difference of the phase delay occurs after the intensity of the reflective laser beam is measured. Additionally, it is difficult to measure the exact radiation intensity due to the differences between the optical modulated waves.

In this paper, in order to solve these problems, we propose a novel method for the measurement of the output of DC voltage that is proportional to the strength of radiation of returned laser beam in the received APD circuit. We therefore implemented a measuring circuit that is able to provide an exact measurement of reflected laser beam. By using the proposed method, we can measure the intensity or strength of radiation of laser beam in real time and with a high degree of precision.

## 2. Optical Sensor

In this section, we describe the basic and application principles of an optical sensor, configuration of industrial optical sensor for high precision, heterodyne technique, current-to-voltage converter and detection of phase difference. 

### 2.1. Basic Principle of an Optical Sensor

Basically, the optical sensor refers to the LDM. The most important parts in the optical sensor are the laser rangefinder and the displacement meter of laser that can be represented as the external interferometer. The optical sensor is basically organized by the laser diode, lens, beam splitter, mirror and photodetector, which are called the optical transducer. This device of the optical sensor can be used to measure the distance to an object by measuring the variation of the frequency, which is the difference of phase displacement of laser beam in the optical frequency, using an optical laser. In other words, the optical sensor measures the distance by measuring the phase difference between the returned signals reflected back from the target and mirror, in which both signals refer to the transmitted modulation signal generated from a laser diode (LD).

Generally, the principle of the LDM can be divide into two techniques: homodyne or heterodyne. When the homodyne technique is used, variation of the original frequency of laser beam and measured frequency is identical. On the other hand, the heterodyne technique uses nonlinearly mixed frequency with an original frequency and the scattered beam with almost close-by constant frequency. Basically, Michelson interferometer and Mach-Zehnder interferometers are the common systems of homodyne and heterodyne systems, respectively.

**Figure 1:**
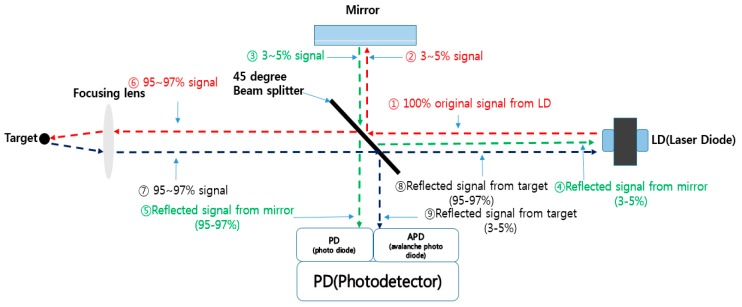
Basic principle of the laser displacement meter (LDM).

Figure 1 shows the basic principle of an optical sensor based on the LDM. The beam (①) that emanates from the LD travels to the target that is the measuring object. Because this beam meets the beam splitter when travelling, the beam is divided into two paths by the beam splitter. Then, they travel separately along two paths that are reflective mirror and target.

One part of the beam (②) (typically less than 3% to 5% of the total beam generated from LD) is selected for one path and sent to the reflective mirror through the beam splitter. This beam approaches the reflective mirror and is then reflected by the mirror so that it is returned again to the beam splitter. When the returned reflective beam (③) from the reflective mirror meet again at the beam splitter, this beam splitter also has same function; it divides the reflective beam into two directions, LD and photodetector. Among the total returned beam from the reflective mirror (③), about 95% to 97% (④) of the strength of radiation travels to photodetector and rest (3% to 5%) (⑤) travels to LD through the beam splitter. Because this returned signal to the photodetector, which is called photo diode (PD), is used as a reference to measure the beam returned from another direction, we call this the “reference signal beam”.

Another part of the beam, involving most of the beam (⑥) (typically more than 95% to 97% of the total beam generated from LD), operates quite similar to the processing in the reflective mirror used as reference signal. This beam goes to the target through the beam splitter and focusing lens from the original LD and is also reflected off the target. Then, this beam is returned (⑦) again to the beam splitter through the focusing lens and is also separated into photodetector and LD. When this beam is reflected off the target, it is scattered into the surrounding area. Basically, this returned beam has to be collected into the focusing lens from the target. Because the beam is scattered, the focusing lens cannot collect the entire beam, and as a result, the strength of the radiation of the beam will be weakened and not constant. Thus, these behaviors cause the generation of error. Among the total returned beam from the target, about 95% to 97% (⑧) of the strength of radiation travel to LD and about 3% to 5% (⑨) of the strength of radiation travel to another photodetector, which is called the “avalanche photo diode” (APD). Thus, we call this “target signal beam” or “measuring signal beam”.

The two returned signal beams (⑤, ⑨) arrive in the photodetector, which is organized by PD and APD. These two returned signal beams have a phase difference between reference signal beam and target signal beam due to a time delay according to the distance differences between the mirror and object. With two returned signal beams (⑤, ⑨), we can determine the distance through the calculation of the phase differences between the reference signal beam (⑤) that comes from the reflective mirror and the reflected target signal beam (⑨) that comes from the target in the photodetector. However, when the laser beam is reflected through the target, the laser beam is scattered and the intensity of radiation will be reduced. As a result, the strength of radiation that arrives at the photodetector is different according to the kind of surface, shape and material media of the reflective target, which causes an error in the distance measured by the optical sensor.

### 2.2. Configuration of Industrial Optical Sensor for High Precision

**Figure 2:**
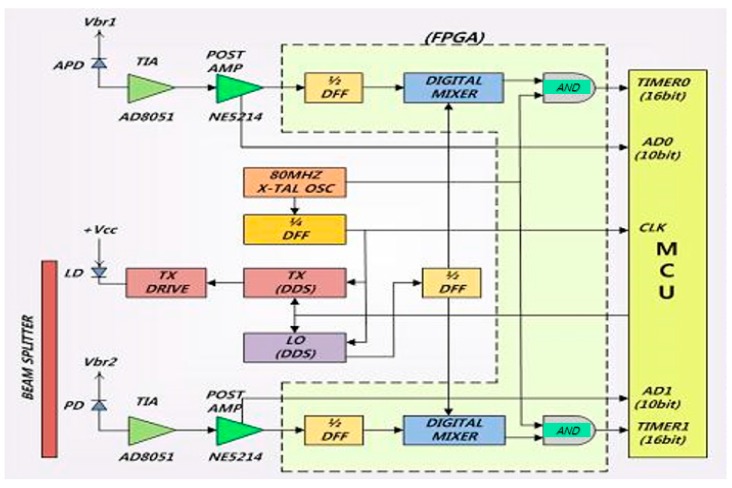
Basic configuration of the industrial optical sensor with high precision.

Figure 2 shows the configuration of an industrial optical sensor based on LDM for high precision. It consists of a field programmable gate array (FPGA) including the digital mixer, a beam splitter, APD, LD, PD, pre-amplification part, control part and its connecting microprocessor that controls the entire displacement of the optical sensor.

Two laser beams enter into the digital mixer through APD and PD. The reference signals come into the digital mixer from PD and the measuring (reflective) signal enters into the digital mixer from APD (in Figure 2). The reference signal and measuring signal can describe by Equations (1) and (2), respectively.

(1) $$S_{reference}=\;A\text{ sin }\omega t_{1}$$

(2) $$S_{measur}=\;B\text{ sin }\omega t_{2}$$

From Equations (1) and (2), we can rearrange into Equation (3) through each of the digital mixers.

(3) $$D_{m}=A\text{ sin }\omega t_{1}\times \;B\text{ sin }\omega t_{2}$$

Equation (3) can be rewritten as Equation (4).

(4) $$D_{m}=\frac{AB}{2}\left[\cos\left(\omega t_{1}-\omega t_{2}\right)-\cos(\omega t_{1}+\omega t_{2}\right)]$$

From Equation (4), we can get a 3-kHz frequency by adjusting A and B of the amplitude coefficient. Because coefficient A is fixed and coefficient B is variable, coefficient B affects Equations (3) and (4). Hence, we get the necessary reflective signal in the LDM as controlling B amplitude.

However, in this case, the reflected signal level and phase difference in the target is not constantly reflected according to the strength of radiation of laser beam; thus, variation caused by reflected signal level of laser beam take place according to the strength and amount of radiation of the laser beam. In accordance with this variation, the error for result of measurement in LDM occurs. The variation caused by the reflected signal level can largely be divided into two types. One is variation by surface re-flexibility in the target with identical distance. The other is that the variation for difference of distance exists in the target, even though they have the same surface re-flexibility. Therefore, we require a compensating technique for the error by the variation of reflective signal level of laser beam in the industrial optical sensor.

### 2.3. Heterodyne Technique

**Figure 3:**
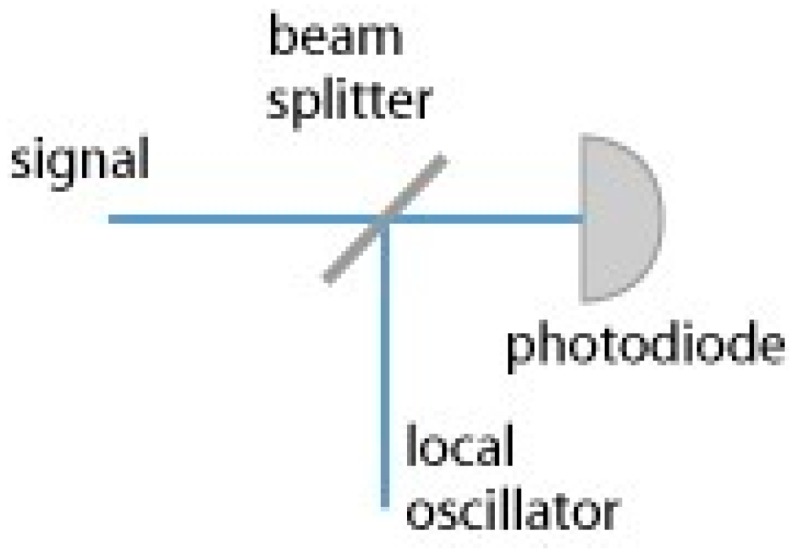
Heterodyne technique.

The heterodyne technique uses the heterodyne-detection principle, whereby a nonlinear optical process—its frequency is nonlinearly mixed with a reference signal from a local oscillator—is used. Figure 3 shows the basic heterodyne technique that consists of a beam splitter and a local oscillator (LO).

The laser beam modulates the frequency signal that is proportionate to the maximum measurement distance, and the reflected beam from the target is then demodulated in heterodyne. The heterodyne can measure the distance after it uses the original modulated signal to detect the phase difference.

The modulated frequency that is proportionate to the maximum measurement distance is represented by Equation (5), as follows:

(5) $$\;f_{m}=\frac{c}{2d}\text{ Hz}$$

where c is the speed of beam $3\;\times \;10^{8}\;m/s$ and d is the maximum measurement distance.

When we directly detect the phase difference between the original modulated signal and the demodulated signal, the error range is extended. We therefore need to transform the frequencies of the two signals into a lower frequency that makes it easy to measure the phase.

The lower frequency is called the “intermediate frequency” and can be represented by Equation (6), as follows:

(6) $$\;f_{i}=f_{l}\pm f_{m}$$

where $f_{l}$ is local oscillation frequency and $f_{m}\;$ is maximum frequency.

We can acquire the intermediate frequency in the demodulated circuit at any time after the local oscillation frequency ($f_{l})$ is found. In this paper, we use the modulated frequency that transforms the same frequency, and the intermediate frequency ($f_{i})$ serves as a reference signal.

Let the reference $f_{ref}$ equal the intermediate frequency as in Equation (7), and let the ratio of the frequency divide (N) be obtained according to Equation (8), as follows:

(7) $$f_{ref}=f_{i}$$

(8) $$N=\;\frac{f_{m}}{f_{i}}$$

### 2.4. Current-to-Voltage Converter

**Figure 4:**
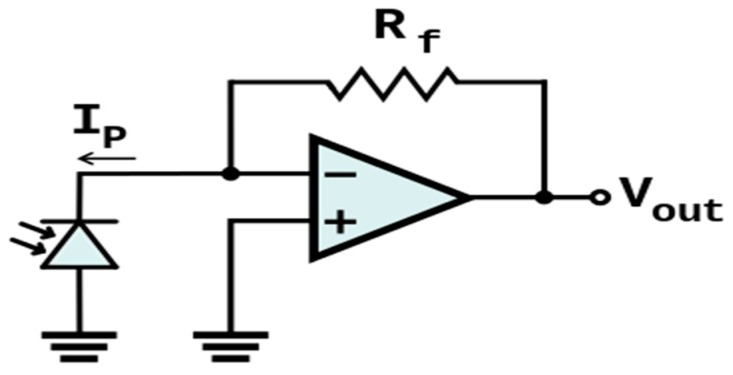
Basic circuit of the trans-impedance amplification (TIA).

There are two methods for the amplification of the signal of modulated weak laser beam. One is a simple voltage-amplification method, and the other is the current-to-voltage-converter method that is also called “trans-impedance amplification” (TIA). The simple voltage-amplification method has the disadvantage of linear characteristics of output voltage compared to the TIA method. The TIA transforms the current signal into a voltage signal using an operational amplifier; Figure 4 shows a basic TIA circuit. Generally, the TIA can be used to amplify the photo detectors to a usable voltage in the optical sensor. Because current-to-voltage converters that are used in the TIA have a current response that is linear to the voltage response, it is more popular to use than a simple voltage-amplification method. Thus, in this paper, we adapted the TIA method.

**Figure 5:**
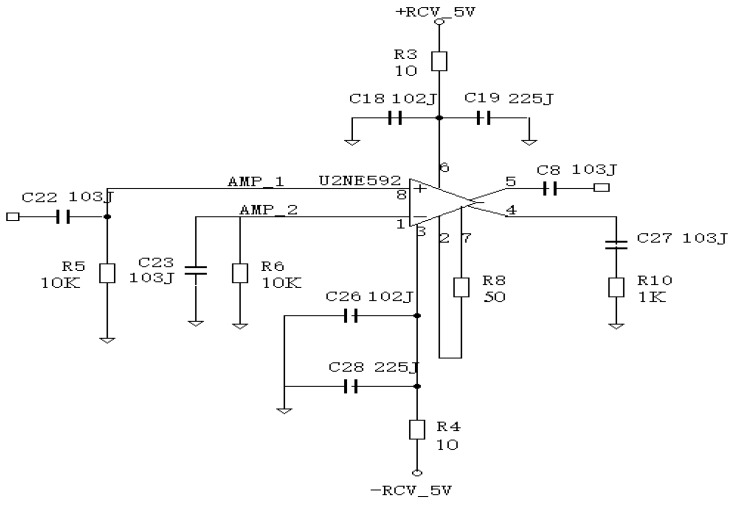
Modified circuit of the TIA from Figure 4.

For this paper, we implemented a modified TIA for which the basic circuit of Figure 4 was used, as is shown in Figure 5.

From Figure 5, we can arrange an input resistance and frequency by following Equations (9) and (10), respectively.

(9) $$\;R_{IN}=\frac{V_{IN}}{I_{IN}}=\frac{R_{F}}{1+A_{vol}}$$

(10) $$f_{-3dB}=\frac{1}{2\pi R_{IN}C_{IN}}$$

The dynamics range is represented by Equation (11) as follows:

(11) $$De=\frac{M}{P}$$

where *M* and *P* represents maximum input current and peak noise current, respectively.

### 2.5. Detection of Phase Difference

To detect the phase difference, we used Equation (12), as follows:

(12) $$\frac{T_{x}}{2}\;\times D_{M}$$

where $T_{x}$ is the phase difference with the reference signal and $D_{M}$ is the distance of maximum measurement. The precision of $T_{x}$ and $D_{M}$ are dependent upon the counter clock and the wavelength of the modulated signal, respectively.

## 3. Measurement Circuit for Strength of Radiation of Reflective Laser Beam

In this section, we describe an error source, principle of measurement, measurement circuit for strength of radiation of returned laser beam and then explain the experimental result and review. 

### 3.1. Error Source

The reasons for errors in the industrial optical sensor based on the LDM are numerous and various. Many researchers carry out research focused on reduction of errors in the industrial optical sensor. There are many sources of errors in the optical sensor such as statistical errors, drift errors, cyclic errors, alignment errors and slope errors. Among these errors, the alignment error is caused by the dependence of the measurement offset upon the width, position, and strength of radiation of the returned beam incident upon the focusing lens. Generally, the biggest error in the optical sensor based on LDM is the measurement error by the difference of phase delay of APD. This error caused by the phase delay occurring in the APD′s output signal according to the strength of radiation of the returned beam at the focusing lens through the target. Nevertheless, this error is very large, and we cannot make any effort to reduce this error as well as measure the error because the exact measuring circuits have not been developed.

In order to measure the error, we have to compensate the difference of phase delay. However, as the optical demodulated signals for measuring are different from each other at the focusing lens, it is hard to measure the strength of radiation of reflective beam.

In this paper, to solve this problem, we need to develop a circuit that can measure the precise reflective beam from the target. Therefore, we propose a novel precise measurement circuit of the reflective beam based on when we measure the output of DC that is proportional to the strength of radiation in the receiving circuit of APD. We can measure the strength of radiation in real time.

### 3.2. Principle of Measurement for Strength of Radiation of Returned Laser Beam

**Figure 6:**
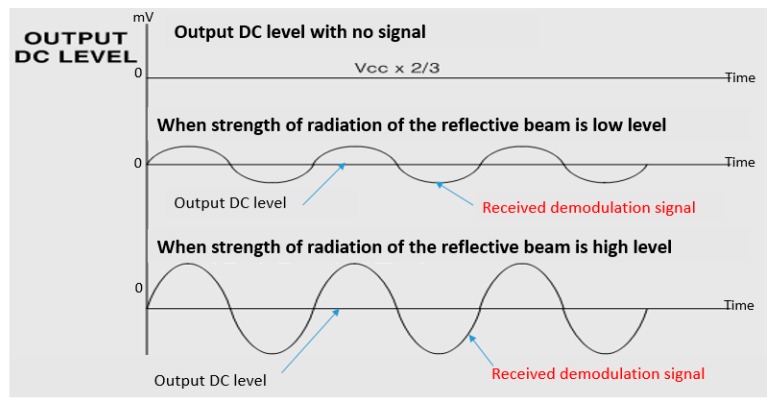
Principle of measurement for the strength of radiation of reflective beam.

When the strength of the radiation of the laser beam that is returned from the target is high, the DC output voltage of the APD receiving circuit is low and the maximum value of the received modulated signal becomes large. Conversely, when the strength of radiation of the reflective beam is low, the DC output voltage of the APD received circuit is high and the maximum value of the received modulated signal becomes small. These principles are shown in Figure 6. 

Generally, the maximum value of a real received modulated signal has only about 4% for the value of output DC level. In Figure 6, however, the output signal of the received modulated signal is large compared to the magnitude of the output DC level for an easier understanding process.

### 3.3. Measurement Circuit

Although a very similar method for measurement of the strength of radiation of the reflective laser beam [25] was proposed, this method does not always provide precise measuring results: it depends on noise and weak signal. Because this method does not have a preprocessing method for DC output voltage including filtering for noise reduction or amplification for magnifying weak signals, it cannot constantly measure the strength of the radiation of the reflective laser beam. 

**Figure 7:**
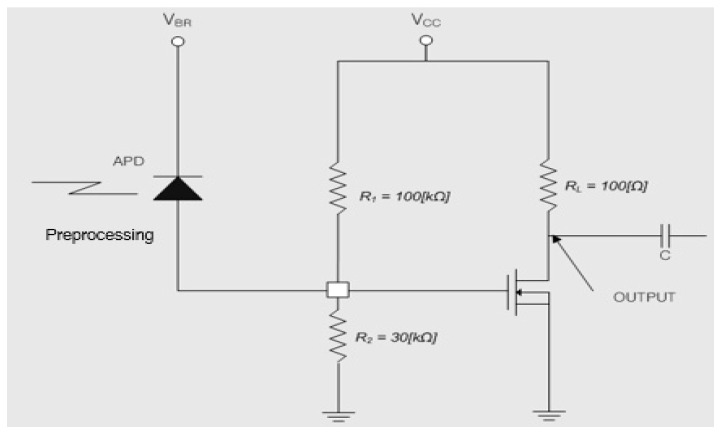
Measurement circuit for measuring the strength of radiation of reflective laser beam.

**Figure 8:**
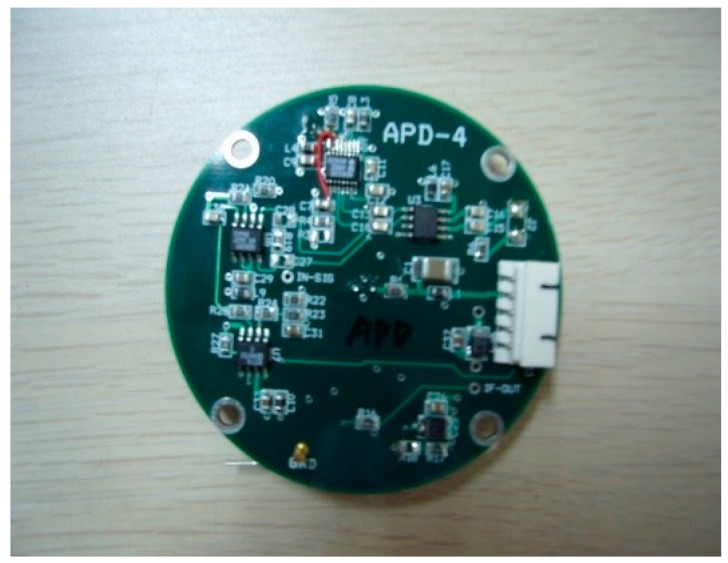
Implemented circuit for measuring the strength of the radiation of the reflective laser beam based on Figure 7.

By using the measurement principal without preprocessing method [25], we proposed an improved amplification circuit with MOSFET with preprocessing method for DC output voltage and we also implemented it as the circuit of measurement for the strength of radiation of the reflective laser beam, as shown in Figure 7 and Figure 8.

### 3.4. Experiments and Review

The amplification circuit in Figure 7 and Figure 8 are composed of a combination that comprises the function of an I-V converter and an amplification circuit with a high gain. The output current of the APD is emitted as the MOSFET output voltage and the gain is approximately 60 dB.

In this paper, when there is no the strength of radiation of the reflective laser beam, we set the DC voltage of the drain of the MOSFET ($V_{d}$), as follows (Equation (13)):

(13) $$V_{d}=V_{cc}\times \frac{2}{3}$$

**Figure 9:**
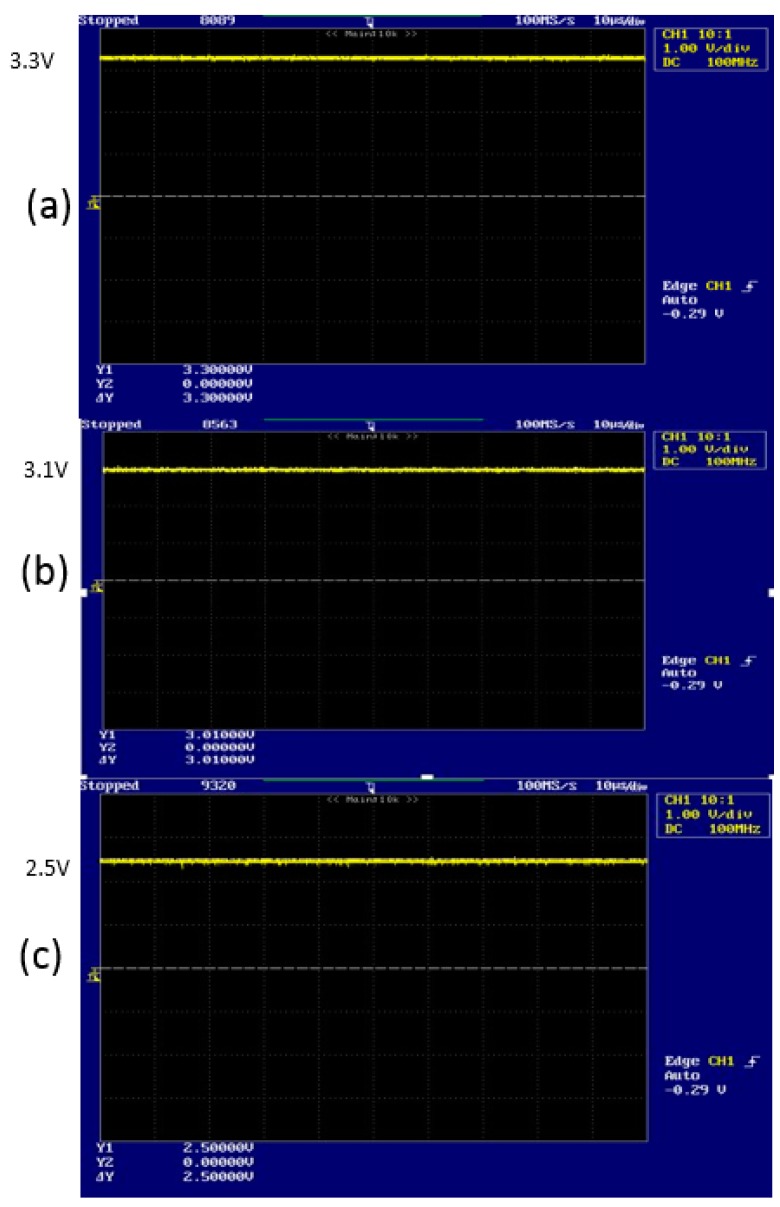
DC-offset output voltage shown as: (**a**) DC 3.3 V; (**b**) DC 3.1 V; and (**c**) DC 2.5 V, respectively, for when the strength of radiation of reflective laser beam are: (**a**) none; (**b**) weak; and (**c**) strong.

In order to satisfy the condition of Equation (13), we set the resistor$\;R_{1}=100\text{ k}\Omega$ and $R_{2}=30\text{ k}\Omega$ and we also set $R_{L}=100\text{ k}\Omega$ so that the value of the drain current $I_{d}$ flows 15 mA to 20 mA, as shown in Figure 8 and Figure 9.

In this experiment, we obtained$V_{cc}=\;+5\text{ V},\;I_{d}=17\text{ mA},\text{ and }R_{L}=100\;\Omega$. When there is no strength of the radiation of reflective laser beam, the drain voltage ($V_{d})$ was measured as 3.3 V.

The relationship of the gate current and the output voltage is described in Equation (14), as follows:

(14) $$\frac{\;V_{d}}{I_{g}}=60\text{ dB},\;\left(G=1000\right)$$

When $I_{g}$ is ±1 μA, we get that $V_{d}$ is 1 mV. Typically, because the dynamics range of APD is ±20 μA to 50 μA, $V_{d}$ becomes ±20 mV to 50 mV. However, the strength of radiation of laser beam transforms the APD-output offset current because the measuring beam of LDM applies to direct optical modulation. As a result, the APD offset current coincides exactly with the strength of radiation of the incident beam. We know that the offset currents that are separated by modulated-signal variation, appear proportional to the intensity of the incident beam at MOSFET $V_{d}$*.*

### 3.5. Experimental Result

Figure 9 represents the output-voltage level that is acquired through the implemented circuit for measuring the strength of radiation of reflective laser beam (Figure 8). Figure 9 shows the DC offset voltage of the drain that as (Figure 9a) DC 3.3 V, (Figure 9b) DC 3.1 V and (Figure 9c) DC 2.5 V when there is (a) none, (b) weak and (c) strong strengths of radiation of reflective laser beam.

**Figure 10:**
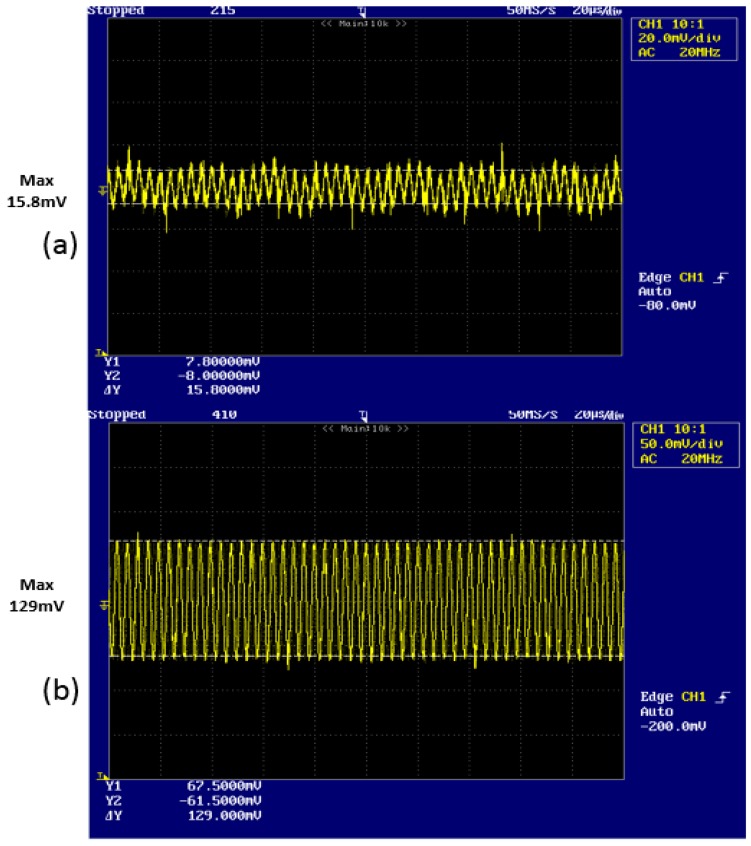
Output signal of received modulated wave with frequency 250 kHz, and maximum voltage: (**a**) 15.8 mV; and (**b**) 129 mV.

Figure 10 shows the received demodulated output signal that have 250 kHz as frequency and (Figure 10a) 15.8 mV and (Figure 10b) 129 mV as maximum voltage, respectively. 

We recognize that Figure 9 and Figure 10 are an exact match for the computational results from Equations (13) and (14).

### 3.6. Discussion of Experimental Result

From Figure 9 and Figure 10, we recognize that we acquire similar results compared with previous results without preprocessing [25]. The previous method cannot always provide the same quality. However, the proposed method with preprocessing always provides the same results under certain circumstance, such as with noise and weak signal of DC output voltage. Therefore, we expect that the proposed method can be applied in the optical sensor based on LDM with constantly excellent quality. 

As an experimental result with implemented circuit to measure the strength of radiation of the laser beam that is returned from the target, we acquire the following results.

1. The DC offset voltage of the drain is DC 3.3 V when there is no strength of radiation of reflective laser beam.
2. The DC offset voltage of the drain is DC 3.01 V when the strength of radiation of reflective laser beam is weak.
3. The received demodulated output signal has 250 kHz frequency and 8 mV maximum voltage.
4. The DC offset voltage of the drain is DC 2.50 V when the strength of radiation of reflective laser beam is strong.
5. The received demodulated output signal has 250 kHz frequency and 129 mV maximum voltage.

From these results, we know that we can improve the performance of optical sensor.

## 4. Conclusions

In this paper, we proposed a novel method for the measurement of the output of direct current (DC) voltage that is proportional to the strength of radiation of returned laser beam in the received APD circuit. We also implemented a measuring circuit that is able to provide an exact measurement of reflected laser beam. By using the proposed method, we can measure the intensity or strength of radiation of laser beam in real time and with a high degree of precision.

From these results, as discussed in the previous section, we confirm that theoretical and experimental results are identical. This conclusion will provide the reduction of measurement error in the optical sensor based on LDM. Therefore we can apply these results to make a real optical sensor based on LDM.
